# Association of Cardiovascular Autonomic Neuropathy and Distal Symmetric Polyneuropathy with All-Cause Mortality: A Retrospective Cohort Study

**DOI:** 10.1155/2021/6662159

**Published:** 2021-05-28

**Authors:** Orsolya E. Vági, Márk M. Svébis, Beatrix A. Domján, Anna E. Körei, Ildikó Istenes, Zsuzsanna Putz, Szilvia Mészáros, Noémi Hajdú, Magdolna Békeffy, Solomon Tesfaye, Péter Kempler, Viktor J. Horváth, Adam G. Tabák

**Affiliations:** ^1^Department of Internal Medicine and Oncology, Semmelweis University Faculty of Medicine, Budapest, Hungary; ^2^Royal Hallamshire Hospital, Sheffield, UK; ^3^Department of Epidemiology and Public Health, University College London, London, UK; ^4^Department of Public Health, Semmelweis University Faculty of Medicine, Budapest, Hungary

## Abstract

**Background:**

People with diabetic cardiovascular autonomic neuropathy (CAN) have increased cardiovascular mortality. However, the association between distal symmetric polyneuropathy (DSPN) or CAN with all-cause mortality is much less investigated. Thus, we set out to examine the effect of CAN and DSPN on all-cause mortality in a well-phenotyped cohort.

**Methods:**

All diabetes cases (*n* = 1,347) from the catchment area of a secondary diabetes care centre who had medical examination including neuropathy assessment between 1997 and 2016 were followed up for all-cause mortality in the NHS Hungary reimbursement database until 2018. We investigated the association of CAN (Ewing tests) and DSPN (Neurometer) with all-cause mortality using Cox models stratified by diabetes type.

**Results:**

Altogether, *n* = 131/1,011 persons with type 1/type 2 diabetes were included. Of the participants, 53%/43% were male, mean age was 46 ± 12/64 ± 10 years, diabetes duration was 13 ± 10/7 ± 8 years, 42%/29% had CAN, and 39%/37% had DSPN. During the 9 ± 5/8 ± 5-year follow-up, *n* = 28/494 participants died. In fully adjusted models, participants with type 1 diabetes patients with versus without DSPN had an increased mortality (HR 2.99, 95% CI 1.4-8.63), while no association with CAN was observed. In type 2 diabetes, both DSPN and CAN independently increased mortality (HR 1.32, 95% CI: 1.07-1.64, and HR 1.44, 95% CI: 1.17-1.76).

**Conclusions:**

Our results are compatible with an increased risk of mortality in people with type 1 diabetes and DSPN. Furthermore, we report a similarly strong association between DSPN and CAN and all-cause mortality in type 2 diabetes mellitus.

## 1. Introduction

Compared to the general population, both type 1 and type 2 diabetes (T1DM, T2DM) confer a higher risk of cardiovascular complications and all-cause mortality. While guideline-directed control of conventional risk factors improves morbidity and mortality of diabetes [1], it still has an increased risk compared to the background population [2]. As cardiovascular risk factors only partly explain this risk, it is conceivable that other characteristics of diabetes (such as its complications) may also play a significant part.

Cardiovascular autonomic neuropathy (CAN) and distal symmetric polyneuropathy (DSPN) are early complications of diabetes that may be already present in prediabetes [3, 4]. The development of neuropathy is a complex process that involves not only hyperglycaemia but also other metabolic factors (e.g., oxidative stress and polyol pathway) [5].

While DSPN is a disabling diabetic complication that mostly affects quality of life via the development of lower limb ulcers, amputations, and frequent falling [6], autonomic nerve dysfunction is associated with an increased risk of cardiovascular mortality [7]. As CAN and DSPN share several pathophysiological mechanisms, it is likely that DSPN could also be associated with an increased cardiovascular mortality. Furthermore, DSPN through its association with falls and infectious complications could also be related to all-cause mortality. Therefore, we aimed to assess the relationship of CAN and DSPN with all-cause mortality in a retrospective cohort of a well-phenotyped diabetes population that attended a secondary care centre.

## 2. Methods

### 2.1. Setting and Participants

This is a retrospective cohort study including all adult diabetes patients who had a detailed neuropathy examination at the neuropathy laboratory of the 1^st^ Department of Medicine, Semmelweis University in 1997-2016. The institute serves as a secondary referral centre for a suburban area of Budapest, Hungary, with ~100 thousand inhabitants. In addition to an assessment of CAN and DSPN, demographic data, anthropometrics, lifestyles, type and duration of diabetes, current and previous illnesses, and contemporary medications were collected on a standardized data entry form. Using the National Health Service (NHS) Hungary identification number, all participants were followed up for all-cause mortality in the NHS Masterfile until December 2018. This research was approved by the local ethical committee under the number of SE TUKEB 36/2017.

**Figure 1:**
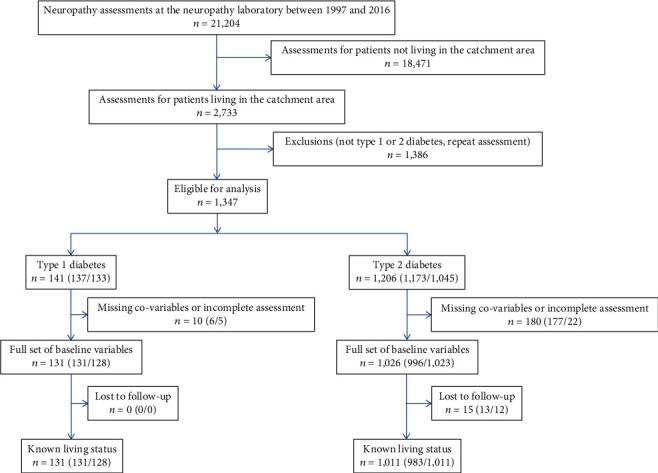
Flow chart of the selection of participants for the current study. Numbers are given for people with any neuropathy. Numbers in brackets are given for people with distal symmetric polyneuropathy/cardiovascular autonomic neuropathy.

To reduce referral bias, we excluded data of people living outside the catchment area of our institution. We further limited the study population to diabetes patients and used the first assessment as the baseline leading to *n* = 1,347 eligible patients. We excluded 7% of T1DM and 15% of T2DM patients due to missing covariates or neuropathy assessments. The mortality follow-up was almost complete (100% for T1DM and 99% for T2DM). The final analytical sample included *n* = 131 (93% of those eligible) T1DM and *n* = 1,011 (84% of those eligible) T2DM patients (Figure 1).

### 2.2. Definition of DSPN and CAN

Subclinical and clinical DSPN (referred as DSPN) and CAN were diagnosed in line with the Toronto Diabetic Neuropathy Expert Group recommendation [6] and were performed using standardized protocols [8]. DSPN was evaluated by Neurometer CPT (Neurotron Inc., Baltimore, USA) [3, 8]. CAN was assessed with gold standard cardiovascular reflex tests using Ewing's battery [6]. See detailed methods in the online appendix (Supplementary material).

### 2.3. Covariables

From the collected information, we derived *sex* and *age* and used the zip code to investigate eligibility. *Height* and *weight* were measured. We defined current smoking as consumptions >1 cigarette/day. Weekly alcohol consumption (beer, wine spirits) was recorded and recalculated as high level of consumption (>14 units/week).

Available medical records were screened for the following medical conditions: type and duration of diabetes, hypertension, myocardial infarction, heart failure, peripheral vascular disease, cerebrovascular accident, dementia, chronic obstructive pulmonary disease, connective tissue diseases, peptic ulcer, liver disease, hemiplegia, chronic kidney disease, and malignancies. This information was supplemented with direct information from the participant.

To estimate comorbidity burden, a simplified Charlson score was calculated by summing the weighted comorbidities. As our study was limited to diabetes patients, information on diabetes, its duration, and neuropathy was not included in the Charlson score [9].

At the time of assessment, a list of *concomitant medications* (trade names) for the last week was requested and coded by the Anatomical Therapeutic Chemical (ATC) classification system.

### 2.4. Outcome

Hungary has a single payer health insurance system that covers most social and health care-related activities. For the current report, all participants were flagged with their NHS ID in the NHS Masterfile, and their last known status was recorded as dead or alive. Follow-up started at the time of neuropathy assessment and was censored at death or inactivation (due to expatriation) or end of follow-up (December 2018) whichever came first.

### 2.5. Statistical Analysis

Based on literature data, the magnitude of the association of DSPN and CAN with all-cause mortality could be within a wide range (hazard ratio [HR] 1.7 to 2.8) in type 1 diabetes [10, 11]. Given the observed mortality in the neuropathy-free population (~15%) and the prevalence of neuropathy (~35%) in our sample of type 1 diabetes, the power was between 68% and 99%. For type 2 diabetes, the reported adjusted hazard ratios were 1.33 to 1.55 that gave excellent power (100%) based on similar calculations to detect differences with similar magnitude to the literature [12, 13].

Given the large age and mortality risk differences between T1DM and T2DM, all statistical analyses were stratified by type of diabetes. Baseline comparisons between participants dead or alive at follow-up were done using 2-sample *t*-tests and *χ*^2^ tests.

We fitted hierarchical Cox proportional hazards models to estimate hazard ratios (HRs) with 95% confidence intervals (CI) for the effects of CAN and DSPN on all-cause mortality. The baseline model (Model 1) was adjusted for age, sex, anthropometric factors (height, BMI), lifestyles (current smoking and alcohol consumption), and diabetes duration. Then, in Model 2, we further adjusted for the presence of hypertension, antihypertensive treatment, and systolic and diastolic blood pressure. The final model (Model 3) was further adjusted for comorbidities (simplified Charlson comorbidity index) and medications (lipid lowering, antianginal, antiarrhythmic, platelet aggregation inhibitors, and anticoagulant medications). First, we run separate models for CAN and DSPN, and finally, we entered both types of neuropathy in a mutual model.

Given the limited number of participants with T1DM, the modelling approach was modified for these persons: we entered potential covariates in the same 3 steps but using a backward stepwise approach.

Sensitivity analyses were conducted to investigate the robustness of our findings. First, we excluded participants with baseline malignancies. Second, we excluded participants if they had died during the first 2 years of follow-up.

All analyses were performed in SPSS version 21.0.

## 3. Results

### 3.1. Baseline Characteristics

Over a mean 9 (SD 5) years of follow-up, 28/131 (21%) T1DM participants died. Persons alive were 12 years younger, leaner, had 4 years shorter duration of diabetes, 5 mmHg lower systolic blood pressure, less likely to have known hypertension and to take antihypertensive or antianginal medications, and had a lower burden of comorbidities (simplified Charlson comorbidity index; all *p* < 0.05). No significant difference in sex distribution, height, lifestyles, diastolic blood pressure, heart rate, and use of other medications was found. While DSPN was less frequent among survivors, the frequency of CAN was similar in the groups (Table 1).

Over a mean 8 (SD 5) years of follow-up, 494/1,011 (44%) T2DM participants died. Similar differences to those in T1DM were found between participants deceased or alive for age, diabetes duration, antianginal medications, and burden of comorbidities. In addition, surviving persons with T2DM were more obese, taller, less likely to consume high amounts of alcohol, had a higher diastolic blood pressure, more likely to be on lipid-lowering medications, and less likely to take anticoagulants (all *p* < 0.05). Furthermore, the risk of hypertension or being on blood pressure-lowering medication was similar in those deceased or alive. Similarly to T1DM, DSPN was less frequent among the surviving persons, while the frequency of CAN was similar in the groups (Table 1).

### 3.2. Association between Neuropathy and All-Cause Mortality in T1DM

According to the Cox model adjusted for age, sex, anthropometrics, lifestyles, and diabetes duration, there was a nonsignificant 16% (HR 1.16 95% CI: 0.50-2.71) increased risk of mortality among participants with CAN at baseline. Given the wide confidence intervals, neither a true effect nor no difference can be excluded (Table 2).

**Figure 2:**
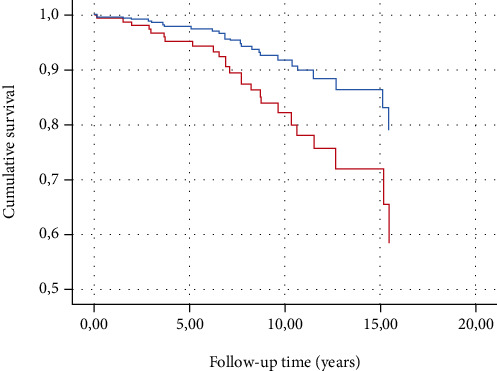
Cumulative survival by DSPN status at baseline in type 1 diabetes patients. Cox proportional hazard model adjusted for age, sex, and current smoking. Curves are fitted for populations with a mean age of 45 years, 53% male, and 36% smoker at baseline. Presence of DSPN at baseline—red line. Absence of DSPN at baseline—blue line. DSPN: distal symmetric polyneuropathy.

According to the results of a similar Cox model, there was a markedly increased risk (HR 2.51 95% CI: 1.00-6.28) of mortality among participants with DSPN at baseline. When the covariables were selected by the backward stepwise method, the results remained (HR 2.99 95% CI: 1.03-8.63) (Table 2 and Figure 2).

### 3.3. Association between Neuropathy and All-Cause Mortality in T2DM

**Figure 3:**
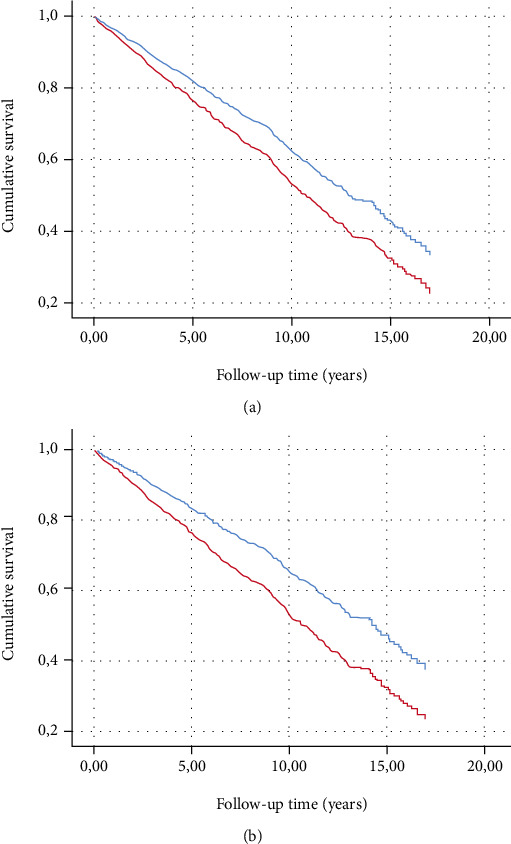
Cumulative survival by CAN (a) and DSPN (b) status in type 2 diabetes patients. Cox proportional hazard model adjusted for age, sex, height, BMI, current smoking, level of alcohol consumption, diabetes duration, known hypertension, systolic and diastolic blood pressure, medications, and burden of comorbidities. Curves are fitted for populations with covariates fixed to the population means. Presence of DSPN/CAN at baseline—red line. Absence of DSPN/CAN at baseline—blue line. DSPN: distal symmetric polyneuropathy; CAN: cardiovascular autonomic neuropathy.

T2DM persons with CAN at baseline had a 31% increased hazard (95% CI: 1.07-1.61) of mortality compared to participants without CAN according to Model 1. This finding remained robust after adjustment for hypertension, blood pressure, medications, and comorbidities. Given that surviving and deceased persons had similar frequencies of CAN, people with CAN should have a strong mortality predictor from Model 1 that explained this finding. Indeed, these people were 4.0 (95% CI: 2.67-5.39) years younger than those without autonomic neuropathy suggesting a selection bias in the referral to our neuropathy service (Table 2 and Figure 3(a)).

The association of DSPN with all-cause mortality was even stronger (HR 1.54, 95% CI: 1.26-1.88) according to Model 1 and remained robust during further adjustments (Table 2 and Figure 3(b)).

To investigate whether the cooccurrence of the two types of neuropathy explained the observed strong associations, a mutual model was built where both CAN and DSPN were entered simultaneously. Effect sizes remained similar in this model, suggesting that the association of CAN and DSPN is mostly independent (Table 2).

### 3.4. Sensitivity Analyses

Both sensitivity analyses confirmed our main analyses showing similar effect sizes for the association between CAN and DSPN and all-cause mortality although the association between CAN and mortality became nonsignificant when we excluded the first 2 years of follow-up (data are available on request.)

## 4. Discussion

Based on the results of a retrospective cohort study from a secondary care centre with an almost complete follow-up, we found a markedly increased risk of mortality in participants with DSPN compared to controls both in T1DM and T2DM during an 8-9-year follow-up. While the confidence intervals for T1DM were wide, it is notable that the point estimate suggests a much stronger association in T1DM (2.51 to 2.99) compared to T2DM (1.54 to 1.44).

In contrast, CAN was not associated with mortality in T1DM, while there was a robust 30% increase in relative risk in T2DM. It should be noted that given the wide confidence intervals in T1DM, an effect similar to that in T2DM cannot be excluded.

According to a model adjusted for potential confounders and mutually including CAN and DSPN among T2DM persons, we found that CAN and DSPN were independent predictors of all-cause mortality.

A meta-analysis of observational studies found a strong association between *CAN* and mortality in both types of diabetes [7].

However, the comparison between our and previous findings in T1DM is hindered by aspects of design and methods of former cohorts. First, we had insufficient power to show modest effects in T1DM, meaning that our null finding is still compatible with even a doubling of risk in participants with CAN (similar to previous findings) [10, 14–18]. Second, the definition of autonomic neuropathy differed in the different cohorts that could largely affect the findings. As there is a clear dose-response association between the numbers of abnormal tests and risk of mortality [7, 15], our definition of CAN as ≥2/4 positive tests also includes “mild” CAN cases. Third, most of these cohorts are coming from secondary and tertiary care centres and thus are prone to selection/referral bias. Lastly, given that mortality predictors can be unequally distributed between participants with and without CAN, only studies that have multiple adjustments can be credibly compared. The point estimates in studies with multiple adjustments are much more homogenous and are in the range of 1.4 to 2.9 [10, 14, 17, 18]. Furthermore, in two of these, the association became nonsignificant in fully adjusted models [14, 18].

The comparisons between the published literature and our findings regarding the association between *CAN* and all-cause mortality are also limited in T2DM [7]. Older studies used one or two Ewing tests to define autonomic neuropathy [19–21], while newer studies used different measures of heart rate variability and QT interval changes with cut-off values not directly comparable to our results [12, 22, 23]. Altogether, these studies showed high relative risks and odds ratios in the range 2-4 in unadjusted analyses [12, 19–23]; however, these estimates were substantially inflated in models adjusted for mortality risk factors to 1.1-1.55 [12, 19–23]. These are close to our estimate of 1.3. These results altogether suggest a modest association between autonomic neuropathy and all-cause mortality after controlling for the effect of conventional risk factors of mortality.

There is strong evidence linking diabetic foot ulcers and all-cause mortality with an almost doubling of risk in both T1DM and T2DM [24, 25]. We found a 2- to 3-times increased all-cause mortality in T1DM with *DSPN* in adjusted models. These findings corroborate and extend those from previous cohorts in multiply adjusted models [10, 11]. While these reports used the vibration perception threshold in addition to absent reflexes and symptoms to define DSPN, we used the current perception threshold as the diagnostic test.

Most studies report an association with point estimates similar to our findings in the range of 1.3 to 2.5 for unadjusted and 1.2 to 1.6 for multiply adjusted models between *DSPN* and all-cause mortality in populations of T2DM irrespective of the diagnostic method [13, 26–29]. Our robust results together with the literature suggest a 30-50% increased risk of death in T2DM with DSPN that is not explained by conventional risk factors.

Diabetic microvascular complications have a similar set of predictors and frequently cooccur [30], suggesting that the effects of DSPN and CAN are not independent. To the best of our knowledge, our study is the first to show that the effect of DSPN and CAN on all-cause mortality is independent not only of conventional predictors but also from each other.

The observation that the association was much stronger between DSPN and mortality in T1DM compared to T2DM seems to be valid and in agreement with literature data. We suspect that this is not a consequence of the different pathophysiologies of T1DM and T2DM but relate to the large age difference between T1DM and T2DM. Younger people in general have better health than older people, and a significant risk factor (such as DSPN) may substantially increase their risk of mortality [31, 32].

A hallmark of CAN is resting tachycardia that is a well-known predictor of cardiovascular mortality [33], QT distance prolongation is frequently found in CAN and may lead to arrhythmias or sudden death [34]. In persons with CAN, symptoms of cardiovascular disease are frequently absent leading to delayed diagnosis and therapy, ultimately resulting in mortality [34]. The disturbed haemodynamic regulation associated with CAN could lead to diabetic cardiomyopathy and could increase the risk of cerebrovascular events [35]. Furthermore, if cardiovascular stressors such as infection or surgery are present, it may increase morbidity and mortality [34]. In addition to cardiovascular events, orthostatic hypotension increases the risk of falls and injuries, another potential cause of mortality [36].

Much less is known about the potential association between DSPN and mortality. In general, the pain caused by neuropathy is thought to be a factor that effects quality of life through disturbed sleep, recreation, and diminished physical and emotional well-being [37]. However, the neuroendocrine, proinflammatory, and neurodegenerative underpinnings of DSPN could also lead to cardiovascular disease, as well as increased oxidative stress and level of advanced glycation end products [38]. DSPN is also a risk factor for medial arterial calcification and balance impairment that could lead to falls and injuries [39]. DSPN is a leading factor of diabetic foot ulcers and amputations, both associated with mortality through infection and chronic inflammation [24, 25].

Alternatively, it is also possible that both CAN and DSPN are markers of other diseases that increase mortality. Indeed, microvascular diabetes complications show remarkable clustering [30]. Diabetic neuropathies may also be markers of a larger cumulative glycaemic exposure. Although we tried to adjust for most risk factors of mortality in participants with diabetes in our analysis, the role of residual confounding cannot be excluded, although the robustness of our findings argues against this.

Our population-based study sample may be representative of persons with diabetes seen in secondary care centres. The large sample size and long follow-up gives sufficient power to investigate even moderate associations between diabetic neuropathies and all-cause mortality. Our study benefits from the use of gold standard measures of diabetic neuropathies. Most important risk factors were collected at baseline that allow the investigation of diabetic neuropathies on top of established risk factors. The use of NHS data allows almost complete follow-up. The fact that all investigations were done using the same methodology in T1DM and T2DM allows comparisons of risks between diabetes types. The fact that our results were robust for adjustments and that the sensitivity analyses were confirmatory also supports the validity of our findings.

Our study has limitations that must be acknowledged. In spite the large number of participants, statistical power within T1DM is limited. Furthermore, as persons with T1DM and T2DM had different risk factor profiles, we were unable to include both types in the same model, limiting the interpretation of these comparisons. As our cohort includes a referred population, referral bias cannot be excluded. Indeed, unadjusted models showed no difference in the prevalence of CAN between deceased and surviving participants. However, we think that our adjusted models represent true differences. It is also likely that the included population has good generalizability to secondary care centres. While our measure of DSPN is noninvasive and probably identifies people with subclinical disease, it imperfectly correlates with the gold standard physiological measures and misses information on signs and symptoms of neuropathy [40, 41]. It should be noted that unless the imprecision of our measurement is directly related to all-cause mortality, it would bias our estimates toward the null. It should also be mentioned that most large-scale observational studies that investigated the association between DSPN and mortality did not use a comprehensive investigation for the diagnosis of DSPN [11, 42]. Our dataset is missing some potentially important confounders and risk factors of all-cause mortality, such as blood tests (lipids, glycaemic measures) and socioeconomic status that makes our results prone to residual confounding. Our results are hypothesis generating only, as we had no data on cause-specific mortality of participants. It could be hypothesized that point estimates could be even higher for those causes that are direct consequences of neuropathy, such as cardiovascular diseases, injuries.

## 5. Conclusion

Our study has clear public health ramifications. We confirmed that CAN is an important predictor of all-cause mortality on top of known cardiovascular and other mortality risk factors. Similarly, DSPN is independently associated with all-cause mortality even in persons without diabetic foot ulcer at baseline. Our results also suggest that the effects of these neuropathies are independent of each other. If these associations are not causal, the presence of any neuropathies still marks an increased risk that should lead to more stringent control of conventional risk factors (such as smoking, lipids, and blood pressure) in persons with neuropathy. If the association is causal, we can hope for better survival if not only symptomatic but etiological treatments become available for diabetic neuropathies. The finding that relative mortality is much higher in persons with sensory neuropathy and T1DM compared to T2DM is novel and suggests that younger age does not protect people from the most severe outcomes although this observation requires further confirmation in other cohort studies.

## Figures and Tables

**Table 1:** Baseline characteristics of participants by type of diabetes and survival status.

	Type 1 diabetes			Type 2 diabetes		
	Alive	Dead	*p*	Alive	Dead	*p*
*n* (%)	103 (78.6)	28 (21.4)		562 (55.6)	449 (44.4)	
Male	53 (51.4)	16 (56.3)	0.672	241 (42.9)	199 (44.2)	0.655
Age (years)	43.1 ± 12	55.9 ± 13	0.001	60.3 ± 9.9	67.4 ± 9.8	0.001
Height (cm)	169 ± 10	168 ± 9	0.879	166 ± 9	164 ± 10	0.002
Weight (kg)	79.5 ± 19.9	79.7 ± 17.8	0.014	86.7 ± 19.6	79.2 ± 15.8	0.001
BMI (kg/m^2^)	27.9 ± 6.4	28.4 ± 6.5	0.006	31.3 ± 5.8	29.5 ± 5.3	0.001
High level of alcohol consumption, *n* (%)	NA	NA	1.000	31 (5.6)	47 (10.4)	0.004
Current smoker, *n* (%)	33 (32.1)	11 (37.5)	0.656	98 (17.5)	81 (18)	0.804
Duration of diabetes (years)	12.5 ± 9.8	16.8 ± 12	0.014	6.8 ± 6.9	7.9 ± 8.5	0.020
Systolic blood pressure (mmHg)	129 ± 16	133 ± 17	0.007	138 ± 17	137 ± 17	0.419
Diastolic blood pressure (mmHg)	80 ± 9	81 ± 10	0.099	81 ± 9	79 ± 9	0.005
Heart rate (beat/min)	78 ± 12	80 ± 12	0.792	76 ± 12	77 ± 14	0.127
Known hypertension, *n* (%)	53 (51.4)	23 (81.3)	0.005	479 (85.1)	387 (86.3)	0.718
Antihypertensive medication, *n* (%)	47 (45.9)	20 (71.9)	0.019	442 (78.7)	347 (77.3)	0.647
Lipid-lowering medication, *n* (%)	16 (15.6)	NA	1.000	221 (39.3)	84 (18.8)	≤0.0001
Antianginal treatment, *n* (%)	NA	NA	0.008	50 (9)	83 (18.6)	≤0.0001
Platelet aggregation inhibitors, *n* (%)	NA	NA	0.097	93 (16.6)	86 (19.2)	0.283
Anticoagulants, *n* (%)	NA	NA	0.115	24 (4.3)	41 (9.2)	0.002
Simplified Charlson comorbidity index	0.4 ± 0.7	1.4 ± 1.1	0.001	1.6 ± 1	2.7 ± 1.4	0.001
DSPN, *n* (%)	34 (33)	17 (60.7)	0.009	202 (36)	193 (42.9)	0.023
CAN, *n* (%)	45 (43.7)	10 (36.7)	0.521	170 (30.2)	139 (30.9)	0.837

Numbers are mean ± SD or *n* (%). *p* values are given for 2-sample *t*-tests or *χ*^2^ tests as appropriate. For cells with *n* ≤ 10, no values are given due to privacy protection regulations. DSPN: distal symmetric polyneuropathy; CAN: cardiovascular autonomic neuropathy.

**Table 2:** The association between CAN and DSPN (Cox proportional hazard models).

	Type 1 diabetes			Type 2 diabetes		
	HR	95% CI	*p*	HR	95% CI	*p*
CAN						
Model 1	1.16	0.5-2.71	0.727	1.31	1.07-1.61	0.009
Model 2				1.29	1.05-1.58	0.016
Model 3				1.33	1.08-1.63	0.007
Mutual model				1.32	1.07-1.64	0.01
DSPN						
Model 1	2.50	1-6.28	0.05	1.54	1.26-1.88	≤0.0001
Model 2				1.53	1.25-1.86	≤0.0001
Model 3				1.49	1.22-1.83	≤0.0001
Mutual model				1.44	1.17-1.76	≤0.0001
Backward stepwise model	2.99	1.04-8.63	0.043			

Model 1: adjusted for age, sex, height, BMI, current smoking, high level of alcohol consumption, diabetes duration; Model 2: as for Model 1+antihypertensive medication, known hypertension, and systolic and diastolic blood pressure; Model 3: as for Model 2+lipid lowering, antianginal, antiarrhythmic, platelet aggregation inhibitor, anticoagulant treatment, and simplified Charlson comorbidity index; Mutual model: as for Model 3 and both autonomic and sensory neuropathy; Backward stepwise model: adjusted for age, sex, and smoking; DSPN: distal symmetric polyneuropathy; CAN: cardiovascular autonomic neuropathy.

## Data Availability

The datasets generated during and/or analysed during the current study are not publicly available due to data protection regulation related to reuse of NHS Hungary data but are available from the corresponding author upon reasonable request.

## Abbreviations

ATC: Anatomical therapeutic chemical
CAN: Diabetic cardiovascular autonomic neuropathy
DSPN: Distal symmetric polyneuropathy
HR: Hazard ratio
NHS: National Health Service
SD: Standard deviation
T1DM: Type 1 diabetes mellitus
T2DM: Type 2 diabetes mellitus.

## Supplementary Materials

Supplementary materials Our paper has supplemental material that contains those details on materials that are only relevant to specialist readers who want to repeat the exact same analysis that we performed but not to the general readership of the journal.
