# Supplementary material for: Association of Cardiovascular Autonomic Neuropathy and Distal Symmetric Polyneuropathy with All-Cause Mortality: A Retrospective Cohort Study
Source: J Diabetes Res. 2021 May 28;2021:6662159. doi: 10.1155/2021/6662159 (PMC8181184; doi:10.1155/2021/6662159)
Supplement: Supplementary materials — Our paper has supplemental material that contains those details on materials that are only relevant to specialist readers who want to repeat the exact same analysis that we performed but not to the general readership of the journal. [file 6662159.f1.docx]

**Supplementary material**

**Methods**

***Definition of sensory and autonomic neuropathy***

All neuropathy examinations were performed by the same assistants according to standardized protocols.[1] Participants were asked to avoid the consumption of caffeine and alcoholic beverages as well as tobacco products at least 12 hours preceding neuropathy testing as well as heavy physical activity on the day before the examination.

Sensory and autonomic neuropathy was diagnosed in line with the Toronto Diabetic Neuropathy Expert Group recommendation.[2] According to the recommendation, the confirmed diagnosis of sensory neuropathy requires at least 2 of the following: the presence of (1) signs or (2) symptoms of sensory neuropathy or (3) abnormal value on a validated measure of small fibre neuropathy. For research purposes, it suggests the use of subclinical sensory neuropathy (the absence of signs and symptoms of neuropathy and abnormal nerve conduction studies or a validated measure of small fibre neuropathy).[2]

Sensory neuropathy was evaluated by the Neurometer CPT (Neurotron Inc., Baltimore, USA) device. Neurometer is an instrument designed for the time-sparing, and non-invasive assessment of clinical and subclinical sensory neuropathy.[3,4,1] The test has adequate diagnostic sensitivity (60-94%) and specificity (95-100%) against gold standard measures of sensory neuropathy if results for the foot are used. [5,6] Similarly, CPT measures of the lower extremity had good to excellent correlations with physical signs and symptoms of sensory neuropathy (r 0.45-0.88) with good to moderate reproducibility.[7,8] Current perception threshold (CPT) was measured at the peroneal nerve (digital branches) by the device at three different frequencies (2 kHz, 250 Hz, 5 Hz).[9] CPT was defined as abnormal if it was outside the following ranges: 1.79-5.23 at 2000 Hz, 0.44 to 2.08 at 250 Hz, and 0.18 to 1.70 at 5 Hz. Sensory neuropathy was defined if there was at least one abnormal CPT result on both sides.

Autonomic neuropathy was assessed with gold standard cardiovascular reflex tests using Ewing's battery [10]. According to current recommendations, the results of the handgrip test were not used [11].The deep breathing test (difference between the highest and the lowest heart rate during expiration over five breathing cycle) was considered abnormal if it was <21.6 for age <20 years, <18.3 for age 20-<30, <15.3 for age 30-<40, <12.9 for age 40-<50, <10.8 for age 50-<60, and <9.1 for age ≥60 years. The RR ratio of postural change (the ratio of RR intervals between the 15^th^ and 30^th^ ventricular beat after standing from lying position) was considered abnormal if it was <1.21 for age <30 years, < 1.13 for age 30-<40 years, <1.05 for age 40-<50 years, <0.99 for age 50-<60 years, <0.91 for age 60-<70 years, and <0.85 for age ≥70 years, The Valsalva ratio (ratio of the longest R-R interval to the shortest R-R interval during forced exhalation) was considered abnormal if it was <1.48 for age <30 years, < 1.39 for age 30-<40 years, <1.32 for age 40-<50 years, <1.25 for age 50-<60 years, <1.17 for age 60-<70 years, and <1.12 for age ≥70 years [12] Orthostatic hypotension (blood pressure differences in response to standing from a lying position) was defined as a blood pressure drop ≥20 mmHg measured after 1-minute or 5-minute standing [2] Autonomic neuropathy was diagnosed if >2 of the above test results were abnormal.

***Demographics, anthropometrics, and lifestyles***

Height was measured to the nearest centimetre, weight to the nearest 0.1 kg in light clothing without shoes using standardized protocols on a digital scale.

***Present and past illnesses at baseline***

*Type and duration of diabetes*

During the abbreviated medical history, information on the type of diabetes, the date of diabetes diagnosis and current antidiabetic treatments were collected from available medical health records and directly from participants. Duration of diabetes was calculated as the date difference between the date of diagnosis and the date of neuropathy examination. Type 1 diabetes was defined as a recording of type 1 diabetes or insulin-dependent diabetes mellitus in the medical record or in the absence of the type of diabetes, diabetes that was diagnosed before 45 years of age and the person was solely on insulin treatment (no other anti-diabetic treatment) at baseline. of type 2 diabetes was defined as diabetes not specified otherwise (e.g. pancreatogenic) and not of type 1 diabetes according to the previous criteria.

*Liver disease*

Liver disease was considered to be mild if liver disease was present but the no mention of cirrhosis was made in the medical health record. Moderate or severe liver disease was diagnosed if liver cirrhosis was reported in the medical health record.

*Chronic kidney disease*

Using the medical history data, we defined severe chronic kidney disease in the presence of uremia or being on dialysis treatment or participants with post-transplant status.

*Simplified Charlson Comorbidity Index*

To estimate comorbidity burden, a simplified Charlson score was calculated for all participants by summing the weighted comorbidities. As all participants without diabetes were excluded from the study and diabetic neuropathy was the major focus of this analysis, information on diabetes, its duration and neuropathy was not included in the Charlson score. The weight for myocardial infarction, congestive heart failure, peripheral vascular disease, cerebrovascular accident (stroke or transient ischemic attack), dementia, chronic obstructive pulmonary disease, connective tissue diseases, peptic ulcer, and mild liver disease was one. The weight for hemiplegia, chronic kidney disease, localized solid tumor, leukemia, and lymphoma was 2, for moderate or severe liver disease it was 3 and for metastatic malignancies it was 6.[13]

***Concomitant medications***

At the time of the neuropathy assessment a full list of concomitant medications (trade names) for the last week was requested and entered into the database. These drug names were translated into codes of the Anatomical Therapeutic Chemical (ATC) classification system. Using these codes the following medication groups were defined: insulin treatment (A10A*), other antidiabetic medications (A10B*), antihypertensive medications (C02*, C03*, C07*, C08*, C09*), lipid lowering medications (C10*), antianginal treatment (C01D*), antiarrhythmic agents (C01A*, C01B*), platelet aggregation inhibitors (B01AC*), and anticoagulants (B01AA*, B01AB*, B01AE*, B01AF*).

***Outcome***

Hungary has a single payer health insurance system that covers most social and health care related activities provided for each citizen. A person’s unique NHS identifier could take different values based on living status and insurance coverage. In addition to the status, the date of status change is also recorded in the NHS Masterfile that allows the generation of status and follow-up time for survival analysis.

For the current report, all participants were flagged with their NHS ID in the NHS Masterfile and their last known status was recorded as dead or alive. Follow-up started at the time of neuropathy assessment and was censored at death or inactivation (due to expatriation) or end of follow-up (December 2018) whichever came first.

As data from death certificates are not routinely entered into the NHS Masterfile, cause of death is only available for those participants who die during a hospitalization and the cause of death is reported to the NHS for reimbursement. Thus cause specific mortality data was not investigated in the current analysis.

**References**

1. Nemeth N, Putz Z, Istenes I, Korei AE, Vagi OE, Kempler M, Gandhi R, Jermendy G, Tesfaye S, Tabak AG et al: Is there a connection between postprandial hyperglycemia and IGT related sensory nerve dysfunction? Nutrition Metabolism and Cardiovascular Diseases 2017, 27(7):609-614.
2. Tesfaye S, Boulton AJ, Dyck PJ, Freeman R, Horowitz M, Kempler P, Lauria G, Malik RA, Spallone V, Vinik A et al: Diabetic neuropathies: update on definitions, diagnostic criteria, estimation of severity, and treatments. Diabetes Care 2010, 33(10):2285-2293.
3. Masson EA, Veves A, Fernando D, Boulton AJ: Curent perception thresholds: a new, quick, and reproducible method for the assessment of pheripheral neuropathy in diabetes mellitus. Diabetologia 1989 Oct., 32:724-8.
4. Putz Z, Tabak AG, Toth N, Istenes I, Nemeth N, Gandhi RA, Hermanyi Z, Keresztes K, Jermendy G, Tesfaye S et al: Noninvasive Evaluation of Neural Impairment in Subjects With Impaired Glucose Tolerance. Diabetes Care 2009, 32(1):181-183.
5. Katims JJ, Naviasky EH, NG LK, Bleecker ML:New screening device for assessment for peripheral neuropathy J Occup Med. 1986 Dec., 28 (12):1219-21.
6. Umezawa S, Kanamori A, Yajima Y, Aoki C: Current perception threshold in evaluating diabetic neuropathy. Diabetes (Japan) 1997, 8:711-9.
7. Rendell MS, Dovgan DJ, Bergman TF, O’Donnell GP, Drobny EP, Katims JJ: Mapping diabetic sensory neuropathy by current perception threshold testing. Diabetes Care 1989 Oct; 12(9):636-40.
8. Tack CJ, NEtten PM, Scheepers MH, Meijer JW, Smits P, Lutterman J: Coparison of clinical examination, current and vibratory perception threshold in diabetic polyneuropathy. Neth J Med. 1994 Feb;44(2):41-9.
9. Pitei DL, Watkins PJ, Stevens MJ, Edmonds ME: The value of the Neurometer in assessing diabetic neuropathy by measurement of the current perception threshold. Diabet Med 1994, 11(9):872-876.
10. Ewing DJ, Campbell IW, Burt AA, Clarke BF: Vascular reflexes in diabetic autonomic neuropathy. Lancet 1973, 2(7842):1354-1356.
11. Korei AE, Kempler M, Istenes I, Vagi OE, Putz Z, Horvath VJ, Keresztes K, Lengyel C, Tabak AG, Spallone V et al: Why Not to Use the Handgrip Test in the Assessment of Cardiovascular Autonomic Neuropathy Among Patients with Diabetes Mellitus? Curr Vasc Pharmacol 2017, 15(1):66-73.
12. Bellavere F, Balzani I, De Masi G, Carraro M, Carenza P, Cobelli C, Thomaseth K: Power spectral analysis of heart-rate variations improves assessment of diabetic cardiac autonomic neuropathy. Diabetes 1992, 41(5):633-640.
13. Charlson ME, Pompei P, Ales KL, MacKenzie CR: A new method of classifying prognostic comorbidity in longitudinal studies: development and validation. J Chronic Dis 1987, 40(5):373-383.
